# The Successful Implementation of a Modified Pediatric Acute Lymphoblastic Leukemia Protocol to Treat an Elderly Patient With T-cell Lymphoblastic Leukemia: A Case Report

**DOI:** 10.7759/cureus.33729

**Published:** 2023-01-13

**Authors:** Ibraheem H Motabi, Yara A Alsaif, Mohammed K Bin Shuayl, Faisal M Farwana, Abdulaziz T Alhassan, Faisal A Alsaif

**Affiliations:** 1 Hematology and Medical Oncology, King Fahad Medical City, Riyadh, SAU; 2 Hematology, King Fahad Medical City, Riyadh, SAU; 3 Orthopedic Surgery, Alfaisal University College of Medicine, Riyadh, SAU; 4 Medicine, Alfaisal University College of Medicine, Riyadh, SAU

**Keywords:** hematological malignancies, treatment of all, children's oncology group, adult t-cell leukemia-lymphoma (atll), t-cell leukemia, acute lymphoblastic leukemia (all)

## Abstract

Acute lymphoblastic leukemia (ALL) is a group of hematological malignancies most commonly seen in pediatrics. The disease process localizes in lymphoid organs, the central nervous system, the mediastinum, and bone marrow (BM). The clinical features of T-cell acute lymphoblastic leukemia (T-ALL) in adults include evidence of generalized lymphadenopathy, hepatosplenomegaly, immunosuppression, and hypercalcemia. There is limited research on the efficacy of using modified pediatric treatment regimens in the elderly over the age of 60 with ALL; this case report aims to illustrate the successful treatment of a 67-year-old male patient diagnosed with T-ALL, using a modified Children's Oncology Group (COG) protocol. Through this, it has been shown to be an effective, safe, and efficacious treatment option for our patient.

## Introduction

Acute lymphoblastic leukemia (ALL) is a group of hematological malignancies derived from B-cell and T-cell progenitors and is characterized by a lack of differentiation, and it has a proliferation of immature lymphoid cells [1]. T-cell acute lymphoblastic leukemia (T-ALL) is a subtype of ALL, which localizes in lymphoid organs, the central nervous system, the mediastinum, and bone marrow (BM) [2]. Among the pediatric population, T-ALL represents 10%-15% of all cases. While in adults, T-ALL accounts for 25% of overall ALL cases [2,3]. The clinical features of T-ALL in adults include evidence of generalized lymphadenopathy, hepatosplenomegaly, immunosuppression, and hypercalcemia [3]. Conventional chemotherapy remains to be the backbone of treatment for ALL [4]. One of the regimens being used was developed by the Children's Oncology Group (COG) and tailored to the pediatric population. It has also been implemented in adolescent and young adult (AYA) populations, which defines patients who were diagnosed with cancer between the ages of 15 and 39 years old [5]. This treatment protocol consists of four phases, which are induction, consolidation, delayed intensification, and maintenance [4]. ALL in older patients often carry genetic alterations that are considered higher risk and typically confer resistance to conventional chemotherapy [6]. There is limited research on the efficacy of using such pediatric treatment regimens in the elderly over the age of 60 with ALL; this case report aims to illustrate the successful treatment of a 67-year-old male with T-ALL, using a modified COG protocol.

## Case presentation

We present a case of a 67-year-old male who is a known case of diabetes and hypertension that presented to the emergency department complaining of cough, shortness of breath, and fatigue. Upon physical examination, there were two palpable cervical lymph nodes. A complete blood count (CBC) was done and showed hemoglobin of 11.2 g/dl, red blood cell (RBC) count of 4.12×10^12^/L, hematocrit of 33.2%, platelet count of 15.4×10^9^/L, and white blood cell (WBC) count of 80.2×10^9^/L. A series of imaging was then ordered, including an anterior-posterior and lateral chest X-ray, which showed homogeneous density in the right lower zone silhouetting the right heart border and right hemidiaphragm. Furthermore, in both frontal and lateral chest radiographs, it showed mediastinal widening. The patient was admitted, and on the following day, a non-contrast chest computed tomography (CT) scan was obtained and showed multiple enlarged mediastinal and abdominal lymph nodes with no bulky lesions, along with moderate right pleural effusion and thickening. In addition, multiple lymph nodes in the upper abdomen with suspicious renal mass and liver lesion were seen. A bone marrow (BM) aspirate showed markedly infiltrated marrow with medium-size blast cells and a high nuclear/cytoplasmic ratio with inconspicuous nucleoli. Cell count was obtained and showed 96% blasts; trilineage hematopoiesis was markedly suppressed. A BM biopsy was also obtained, and it showed hypercellular marrow (100% cellularity), and on the hematoxylin and eosin section, significant infiltration by immature cells was noted.

Peripheral blood flow cytometry was also performed, and the result was that the cells expressed were cluster of differentiation (CD) 5, CD10, CD2, CD7, CD4, CD71, CD1a, CD99, terminal deoxynucleotidyl transferase (TdT), and cytoplasmic (Cy)-CD3 positive. However, they lacked surface (S)-CD3, CD8, human leukocyte antigen (HLA)-DR, and CD34 positivity. Then, a fluorescence in situ hybridization (FISH) analysis was obtained, and the results showed no evidence of *TLX3* and *TCRB* gene rearrangement; there was a signal pattern that was consistent with *TLX1* gene (10q24.31 rearrangement) in 87% of scored nuclei (174/200). Another signal pattern was noted and was consistent with *TCRA/D* gene (14q11.2 rearrangement) in 85.5% of scored nuclei (171/200). The average of those two gene rearrangements was 86.25% of scored nuclei. A diagnosis of T-ALL was established, and the management plan agreed upon was started using the COG protocol for ALL. Our patient received four phases of chemotherapy illustrated in Table 1. During the treatment course, our patient was found to have renal cell carcinoma in the left kidney, for which he underwent a nephrectomy that resulted in dose modifications of his treatment course.

**Table 1:** Illustrating the COG treatment protocol IV, intravenous; IM, intramuscular; PO, orally; BID, two times a day; TID, three times a day; NS, normal saline; PEG, polyethylene glycol; COG, Children's Oncology Group

Treatment phase	Dose	Route	Days
Induction			
Vincristine (capped at 2 mg)	2 mg	IV	1, 8, 15, and 22
Daunorubicin	25 mg/m^2^	IV	1, 8, 15, and 22
L-asparaginase	6,000 units/m^2^	IM	Thrice weekly for three weeks
Prednisolone	30 mg/m^2^ (BID)	PO	1-28
Cytarabine	70 mg	Intrathecal in 10 ml NS	1
Methotrexate	15 mg	Intrathecal in 10 ml NS	8 and 29
Consolidation A			
Cyclophosphamide	1,000 mg/m^2^	IV	1
Mesna	360 mg/m^2^	IV	1
Mercaptopurine	60 mg/m^2^	PO	1-14
Methotrexate	15 mg	Intrathecal in 10 ml NS	1, 8, 15, and 22
L-asparaginase	6,000 units/m^2^	IM	17, 19, 21, 23, and 25
Vincristine (capped at 2 mg)	2 mg	IV	15 and 22
Consolidation B			
Cyclophosphamide	1,000 mg/m^2^	IV	1
Mesna	360 mg/m^2^ (TID)	IV	1
Cytarabine	75 mg/m^2^	IV	1-4 and 8-11
Mercaptopurine	60 mg/m^2^	PO	1-14
L-asparaginase	6,000 units/m^2^	IM	17, 19, 21, 23, and 25
Vincristine (capped at 2 mg)	2 mg	IV	15 and 22
Interim maintenance			
Vincristine (capped at 2 mg)	2 mg	IV	43
Methotrexate	5,000 mg/m^2^	IV	43
Mercaptopurine	25 mg/m^2^	PO	1-56
Delayed intensification A			
Vincristine (capped at 2 mg)	2 mg	IV	1, 8, and 15
Dexamethasone	5 mg/m^2^ (BID)	PO	1-7 and 15-21
Doxorubicin	25 mg/m^2^	IV	1, 8, and 15
PEG-asparaginase	2,500 units/m^2^	IM	4
Methotrexate	15 mg	Intrathecal in 10 ml NS	1
Delayed intensification B			
Vincristine (capped at 2 mg)	2 mg	IV	15 and 22
PEG-asparaginase	2,500 units/m^2^	IM	1
Cyclophosphamide	1,000 mg/m^2^	IV	1
Mesna	360 mg/m^2^	IV	3-6 hours post cyclophosphamide
Cytarabine	75 mg/m^2^	IV	1-4 and 8-11
Thioguanine	60 mg/m^2^	PO	1-14
Methotrexate	15 mg	Intrathecal in 10 ml NS	1 and 28

On follow-up after remission, a chest CT scan was obtained, and it showed cluster of tiny nodules with a tree in bud configuration in both the upper lobe and right middle lobe, likely due to infective etiology. Another CT was ordered to ensure resolution: no enlarged nodes in the chest and small hypodense splenic lesion. Additionally, a post-induction BM biopsy was done and was consistent with remission. Finally, flow cytometry was also performed post-induction on the BM specimen and showed no evidence of residual leukemia.

## Discussion

This report documents a case of T-ALL in a 67-year-old male who was treated successfully using the modified COG protocol resulting in disease remission. When comparing the outcomes of de novo T-ALL to those observed in B-cell acute lymphoblastic leukemia (B-ALL), 85% five-year event-free survival rate improvement is observed in patients with T-ALL. That can be accredited to the new treatment modalities of T-ALL [4]. The overall survival (OS) of patients with T-ALL in pediatric age groups has better outcomes than in adults, with an average five-year OS rate of 80% in pediatrics as opposed to less than 50% in the adult population; this could be accredited to higher treatment related to toxicities seen in adults [7,8]. Historically, patients with B-ALL had better outcomes when compared to patients with T-ALL. However, this is not the case nowadays, thanks to the new T-ALL intensive treatment modalities, with OS of over 90% and a five-year event-free survival rate exceeding 85% [9].

There are multiple modalities being implemented to aid treatment outcomes and further other modalities being studied. One of the treatment modalities used to treat such cases is hematopoietic stem cell transplantation (HSCT); of note, T-ALL patients with persistent minimal residual disease (MRD) can be offered HSCT, which is not usually used in the first remission phase [10]. There are some other drugs that are currently being studied, such as nelarabine and bortezomib [11,12].

Nelarabine is the only drug approved for specific use in relapsed or refractory (R/R) T-ALL/T-cell lymphoblastic lymphoma (T-LBL); however, this is based on limited data [4]. Moreover, the addition of nelarabine to different treatment regimens in children and young adults has shown improved disease-free survival without an increase in toxicity levels [11]. Furthermore, a study about bortezomib has suggested that there is potential for this drug to be an effective treatment through the inhibition of nuclear factor kappa-light-chain-enhancer of activated B cells (NF-κB) activation both in vivo and in vitro, but studies are still being conducted on it [12].

Our patient is a 67-year-old male who was treated using a dose-modified COG protocol, through which he achieved complete remission. Treatment regimens that are implemented in adults are largely derived from pediatric protocols; that being said, the pediatric population still has a better overall cure rate reaching 80% as opposed to the adult population, which demonstrates 30%-40% cure rates in one study, attributed to higher relapse rates and the refractory nature toward conventional chemotherapy [13]. Another study also demonstrated that a markedly improved outcome of adults with ALL was noted when a pediatric-inspired therapy was applied to them [13].

## Conclusions

In summary, ALL defines a group of hematological malignancies, of which T-ALL is a subtype commonly seen among the pediatric population. That being said, when T-ALL occurs in adults, it is a challenging disease to treat, especially in elderly patients. Treatment regimens that are implemented in adults take inspiration from pediatric protocols. Herein, we are reporting a 67-year-old male patient diagnosed with T-ALL who achieved complete remission using a modified COG protocol. Through this, it has been shown to be an effective, safe, and efficacious treatment option.
